# High variability of TB, HIV, hepatitis C treatment and opioid substitution therapy among prisoners in Germany

**DOI:** 10.1186/s12889-017-4840-4

**Published:** 2017-10-25

**Authors:** Jana Müller, Daniel Schmidt, Christian Kollan, Marc Lehmann, Viviane Bremer, Ruth Zimmermann

**Affiliations:** 1 Department of Infectious Disease Epidemiology, HIV/AIDS, STI and Blood-borne Infections, Robert Koch Institute, Berlin, Germany; 2 Charité - Universitätsmedizin Berlin, Berlin, Germany; 3 Head of Medical Services in the Berlin state prison system, Berlin, Germany

**Keywords:** TB, HIV, HCV, OST, Treatment, Prison health, Intramural, Secondary data

## Abstract

**Background:**

In Germany, medical care of prisoners is completely separated from extramural health care. The extent and quality of medical care among prisoners in Germany are therefore largely unknown. We performed a secondary data analysis of pharmacy sales data for tuberculosis (TB), HIV, hepatitis C (HCV) and opioid substitution treatment (OST) delivered to prisons in 11 federal states (FS) in Germany between 01/2012 and 03/2013. The aims of this study were to assess (i) the treatment availability for the selected diseases and OST in German prisons, (ii) the proportion of prisoners treated per FS and overall for TB, HIV, HCV and OST during the study period.

**Methods:**

Substances unique to or typically used for the treatment of each disease were defined as marker substances with defined daily doses (DDD).

For each marker substance we assessed the cumulative number of DDD, the average daily number of DDD (DDD_d_) and average treatment prevalence per day in percent (adTP). Accordingly, the DDD_d_ represents one person treated per day and the adTP means the proportion of prisoners treated per day. We compared the adTP of the diseases with previously measured prevalences.

**Results:**

We obtained data from pharmacies supplying prisons in 11 of 16 German FS. Of the included prisons, 41% were supplied with medicines for TB, 71% for HIV and 58% for HCV and OST. Twice as many delivered marker substances for TB were indicated for the continuation phase and chemoprevention than the intensive phase. The HIV adTP ranged from 0.06% to 0.94%, HCV adTP ranged from 0.03% to 0.59% and OST adTP ranged from 0% to 7.90%. The overall adTP for the respective treatment was 0.39% for HIV, 0.12% for HCV and 2.18% for OST.

**Conclusions:**

According to our findings treatment rates for TB were consistent with the expected TB prevalence, at least in Berlin. HIV treatment seems to be offered to an adequate proportion of estimated infected prisoners. In contrast, the HCV treatment prevalence was low. High variation among FS in provision of all treatments, particularly of OST, point to inconsistent treatment practices, although nationwide extramural treatment guidelines for Germany exist.

## Background

Studies have shown that specific blood- and air-borne and sexually transmitted infections are more common among prisoners than in the general population: in Germany and other European countries, tuberculosis (TB) prevalence was 11 to 81 times higher, hepatitis C virus (HCV) prevalence was 17 to 100 times higher, human immunodeficiency virus (HIV) prevalence was 5 to 24 times higher and opioid dependence was 70 times higher among prisoners in comparison to the general population [1–8]. TB is primarily an airborne disease and the bacteria are usually spread from person to person through infectious droplet nuclei when an infectious pulmonary TB patient coughs or sneezes [9]. Usually, a prolonged and close contact is required for transmission; therefore the prison setting can facilitate the spread of the disease. HIV and HCV can be transmitted via unprotected sexual contacts as well as through the widespread intramural practice of unsafe drug use and tattooing involving the sharing of potentially infectious needles, syringes and other paraphernalia [8, 10–16]. Thus transmission risks and infection events are highly increased in prisons, especially due to the absence of sterile drug injecting utensils and restricted access to condoms and other prevention measures.

Nevertheless, the prison setting presents not only challenges, but also opportunities for the prevention and treatment of TB, HIV and hepatitis [17]. Prevention of HIV and HCV by offering testing and counselling, providing condoms and tattooing materials as well as sterile injection equipment for people who inject drugs (PWID) also includes the initiation and continuation of opioid substitution treatment (OST) to reduce injection frequency. Furthermore, the treatment of newly diagnosed and already known infections is important not only for the person infected but also in terms of treatment as prevention [6, 18–23]. Despite various challenges in providing treatment for the mentioned infectious diseases and offering OST in the prison setting, it is practicable, and crucial to reduce transmission within prisons.

Different screening approaches exist to identify infections in prisons; however, systematic screening for infectious diseases is not implemented in the German prison system. Strategies differ among federal states (FS) and singular prisons. TB screening by chest x-ray is performed systematically on all prisoners at entrance in the FS of Berlin [24], and in some other prisons, but in most FS, symptom-based screening strategies are implemented. Screening for sexually transmitted and blood-borne viruses is also diverse, and ranges from test offer to persons with clinical symptoms or risk factors, request by the prisoner to mandatory testing in some prisons or FS [5].

In Germany, treatment guidelines and effective treatment regimens for TB, HIV and HCV as well as OST are available [25–29], and all mentioned treatments are being carried out among patients with statutory health insurance (SHI) extramurally. Upon incarceration SHI is suspended, and health care is provided and paid for by the federal Ministry of Justice (MoJ) of the respective FS. As a result of this transitional period between health care providers treatment interruptions may occur [14, 30].

During the study period from January 2012 to March 2013, 67.607 people were detained in 186 prisons in the 16 FS of Germany [31], corresponding to nearly 0.08% of the total German population. Throughout the study period, five pharmacies supplied all prison hospitals and prisons in Germany with pharmaceuticals. Implementation and provision of health care lays within the responsibility of the MoJ of the respective FS [32]. Nevertheless, according to national laws (Prison Act § 56ff StVollzG; Social Act SGB V), health care in the penitentiary system should take place under the principle of equivalence of care and within the standards of the SHI [14, 33]. Health care is implemented by the prison doctor with the help of the prison administration, both of whom are under the supervision and directives of the FS [14, 34, 35]. Medical care of prisoners is provided by the prison doctor in out-patient care, in special prison wards and correctional hospitals or wherever necessary by extramural specialized medical doctors or hospitals [14]. Since not every prison has a sick ward and only some FS have prison hospitals, contracts and transfer co-operations exist among the states in order to ensure medical care in every FS [34, 36–38].

Because prison health care is not part of the regular public health system in Germany, it is therefore not part of the health reporting system [14]. The extent and quality of TB, HIV, HCV and opioid dependence treatment provided to prisoners in Germany are therefore largely unknown [5, 6, 39].

In order to determine the medical care of infectious diseases and opioid dependence among prisoners in Germany we performed a secondary data analysis of pharmacy sales data for TB, HIV, HCV treatments and OST delivered to prisons in 11 FS in Germany between January 2012 and March 2013. The aims of this study were to assess (i) the treatment availability for the selected diseases and OST in German prisons (ii) the proportion of prisoners treated per FS and overall for TB, HIV, HCV and OST during the study period.

## Methods

We asked the MoJ of all 16 FS in Germany to approve and support the planned data collection and analysis for each respective FS in August 2013. Twelve FS agreed to participate in the study; however, one FS was excluded because the respective pharmacy did not provide the data. In the participating FS, all prisons and prisoners of the respective FS were included except one sick ward (5 beds) and one correctional hospital (52 beds) because they were not supplied by one of the contract pharmacies. Throughout the study period, all participating prisons and prison hospitals were supplied by three pharmacies with TB, HIV, HCV and OST medicines.

The pharmacies provided the data for the period from 01/2012 to 03/2013. The dataset contained a minimum of eight variables: the name of the prison, the FS, the trade name of the drug, package size, dosage form, the Anatomical Therapeutic Chemical (ATC) classification code of the drug, the central pharmaceutical number (*Pharmazentralnummer, PZN*), and the number of drug packages supplied per month. The study collected solely prescription data and no individual patient data. No ethical or data protection concerns were raised. The names of the prisons were pseudonymized.

Substances unique to or typically used for the treatment of each disease were defined as marker substances for the respective disease. We used defined daily doses (DDD) of the marker substances to calculate the number of daily treated persons. The DDD were determined based on current national treatment guidelines, prescribing information according to the German Medicines Act and literature research (Table 1). The number of standard units (e.g. tablets, pens) was determined for each marker substance.

**Table 1:** Marker substances and DDD

Disease	Marker substances	DDD [mg]
Tuberculosis [26, 58]	Ethambutol (E)	1200
	Pyrazinamid (Z)	1500
	Isoniazid (H)	300 ^a^
	Rifampicin (R)	600
	Protionamide (Pto)	750
	Terizidone (Trd)	750
	Rifabutin (Rfb)	150
Hepatitis C [27, 58–60]	Pegylated interferon-α (PEG-INF)	0.05, 0.08, 0.1, 0.12, 0.135, 0.15, 0.18
	Boceprevir (BOC)	2400
	Telaprevir (TVR)	2250
HIV [25, 58]	Emtricitabin (FTC)	200
	Lamivudin (3TC)	300
Opioid dependence [29, 61–64]	Methadone,	90
	Levomethadone	45
	Buprenorphine	8
	Buprenorphine/Naloxone	8

^a^For the formulation of 400 mg isoniazid per pill the determined DDD was 400 mg

**Fig. 1:**
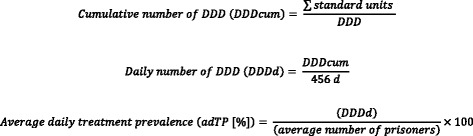
Cumulative number of DDD, average daily number of DDD and average daily treatment prevalence

First, we assessed the cumulative number of DDD (DDD_cum_) of the marker substances for the whole study period (456 days). Then we calculated the average daily number of DDD for each marker substance for the study period (DDD_d_). Accordingly the DDD_d_ represents one person treated with the respective substance per study day. Finally, we calculated the average treatment prevalence per day in percent (average daily treatment prevalence, adTP). Accordingly, the adTP means the proportion of prisoners treated per day with the respective drug to the average number of all prisoners during the study period (Fig. 1). We compared the adTP with previously measured prevalences.

**Fig. 2:**
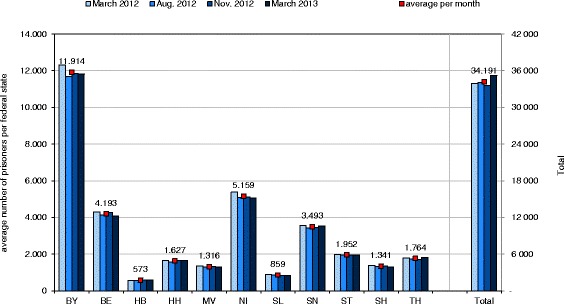
Average number of prisoners per month in the respective FS and in total during the study period January 2012 and March 2013. Bavaria (BY), Berlin (BE), Bremen (HB), Hamburg (HH), Mecklenburg-Western Pomerania (MV), Lower Saxony (NI), Saarland (SL), Saxony (SN), Saxony-Anhalt (ST), Schleswig-Holstein (SH) and Thuringia (TH)

The number of incarcerated persons was obtained from the German Federal Statistical Office, which provides this data in March, August and November each year [31]. Based on these data an average monthly number of prisoners for the months of March 2012, August 2012, November 2012 and March 2013 were calculated for each participating FS and for all participating states in total (Fig. 2).

### TB treatment

A standard six month treatment regimen for TB consists of the four antitubercular substances ethambutol (E), pyrazinamid (Z), isoniazid (H) and rifampicin (R). Patients receive all four drugs daily for the first two months (intensive phase), followed by H and R daily for another four months (continuation phase). The marker substances for anti-TB standard treatment were determined to be E, Z, H and R. The determined DDD were 1200 mg for E, 1500 mg for Z, 300 mg for H (except for the formulation 400 mg per pill) and 600 mg for R as recommended in the ATC classification (Table 1). The standard regimen for latent tuberculosis infection (chemoprevention) consists of either (i) H alone or (ii) a combination of H and R or (iii) R alone [26]. For chemoprevention the marker substances were determined to be H (DDD 300 mg) and/or R (DDD 600 mg). R and H fixed-dose combinations were divided into single substances. Pyridoxin as an additive to H was not taken into account. For multidrug-resistant-TB (MDR-TB), protionamide (Pto) and terizidone (Trd) were determined to be the marker substances with a DDD of 750 mg. For HIV-TB-coinfection the marker substance was rifabutin (Rfb) with a DDD of 150 mg (Table 1).

### HIV treatment

The standard therapy for HIV during the study period contained exactly one thiacytidine medication (TCM), either lamivudine (3TC) or emtricitabine (FTC). The marker substances for HIV treatment were determined to be 3TC and FTC (Table 1) [25, 40, 41]. The determined DDD were 300 mg for 3TC and 200 mg for FTC. Drugs with more than one substance were split into single substances.

### HCV treatment

The standard therapy for HCV during the study period consisted of peginterferon α-2a (PEG-IFN α-2a) or peginterferon α-2b (PEG-IFN α-2b) in combination with ribavirin (RBV). Furthermore, during the study period a triple-therapy with the substances boceprevir (BOC) or telaprevir (TVR) in combination with PEG-IFN and RBV was available. The marker substances for HCV treatment were determined to be PEG-IFN α-2a, PEG-IFN α-2b, BOC and TVR. We assumed that one pen PEG-IFN correlated with one treated person. The determined DDD were 2400 mg for BOC and 2250 mg for TVR (Table 1).

### OST

The marker substances for OST were determined to be methadone, levomethadone, buprenorphine and buprenorphine/naloxone. The determined DDD were 90 mg for methadone, 45 mg for levomethadone, and 8 mg for buprenorphine and buprenorphine/naloxone (Table 1).

## Results

**Fig. 3:**
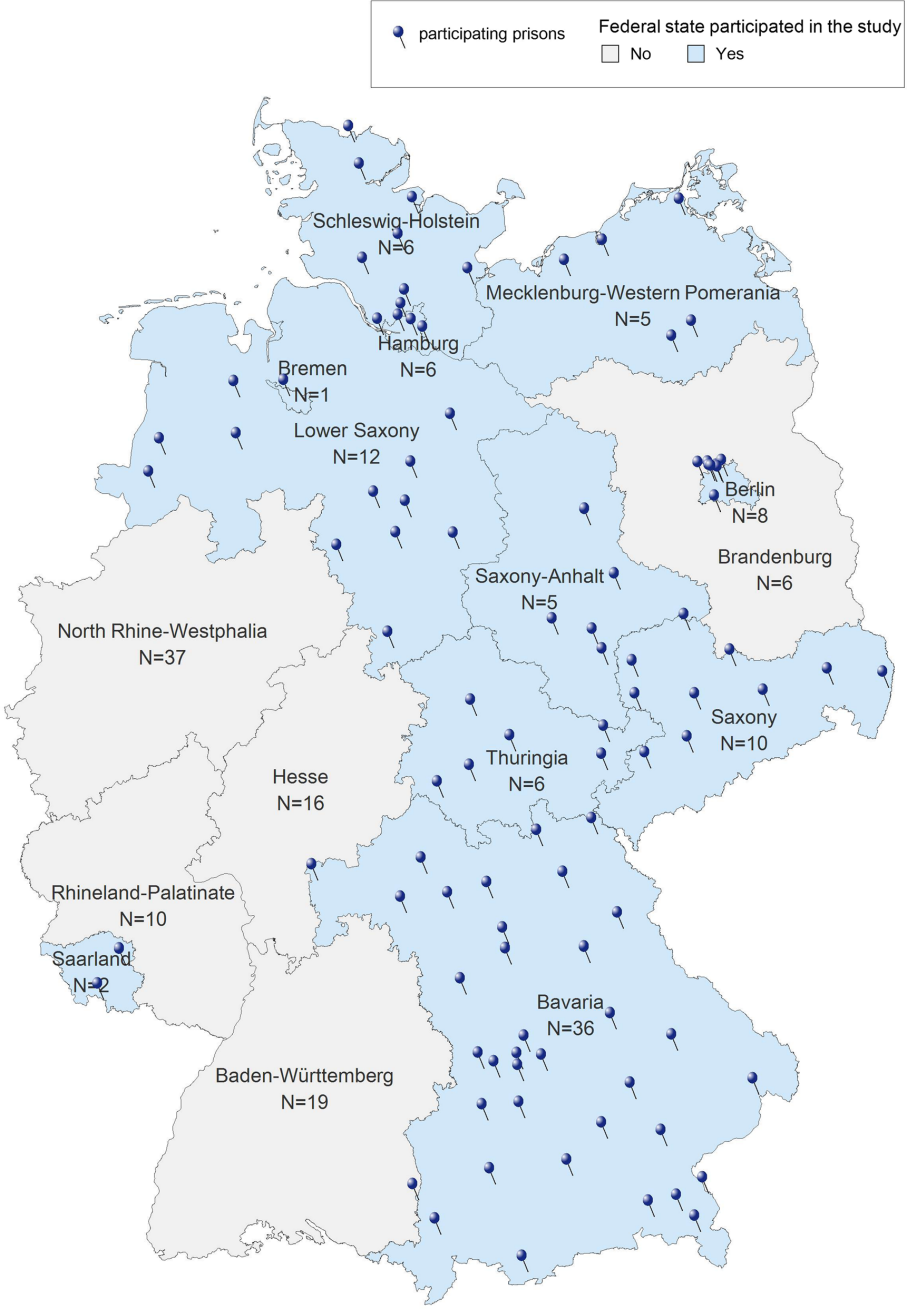
Participation in the study and number of prisons in the respective FS of Germany

By June 2014, of the total 16 German FS, the MoJ of the 12 FS Bavaria, Berlin, Bremen, Hamburg, Mecklenburg-Western Pomerania, Lower Saxony, Rhineland Palatinate, Saarland, Saxony, Saxony-Anhalt, Schleswig-Holstein and Thuringia had agreed to the study. Rhineland Palatinate could not deliver the data and was excluded. In the study period the 11 participating FS with 34,191 prisoners in 97 prisons represented almost half of all German prisoners (*N* = 67,607) in 186 prisons (Fig. 3).

Detailed results for each disease and FS are shown in Table 2.

**Table 2:** Number of prisons, number of delivered prisons, average number of prisoners, cumulative number of DDD (DDD_cum_), average daily number of DDD (DDD_d_) and average treatment prevalence per day in percent (adTP)

Federal state	Bavaria	Berlin	Bremen	Hamburg	Mecklenburg-Western Pomerania	Lower Saxony	Saarland	Saxony	Saxony-Anhalt	Schleswig-Holstein	Thuringia	Overall
Number of prisons	36	8	1	6	5	12	2	10	5	6	6	97
Average number of prisoners (03/2012, 08/2012, 11/2012, 03/2013)	11,914	4193	573	1627	1316	5159	859	3493	1952	1341	1764	34,191
Number of prisons with delivery of TB drugs	13	3	1	4	2	9	0	2	3	3	3	40
H DDD_cum_	3636	2484	251	1877	167	1734	0	100	2351	500	333	13,432
R DDD_cum_	4640	2455	293	1633	100	1050	0	140	790	85	0	11,185
E DDD_cum_	2037	1440	135	1209	0	271	0	242	42	0	104	5480
Z DDD_cum_	1653	1965	134	1101	0	300	0	67	100	0	0	5319
Pto DDD_cum_	0	100	0	0	0	0	0	0	0	0	0	100
Trd DDD_cum_	251	284	0	0	0	0	0	0	0	0	0	534
RFB DDD_cum_	405	0	0	0	0	0	0	0	0	0	105	510
H DDD_d_	8	5	1	4	0	4	0	0	5	1	1	29
R DDD_d_	10	5	1	4	0	2	0	0	2	0	0	25
E DDD_d_	4	3	0	3	0	1	0	1	0	0	0	12
Z DDD_d_	4	4	0	2	0	1	0	0	0	0	0	12
Pto DDD_d_	0	0	0	0	0	0	0	0	0	0	0	0
Trd DDD_d_	1	1	0	0	0	0	0	0	0	0	0	1
RFB DDD_d_	1	0	0	0	0	0	0	0	0	0	0	1
H adTP	0.067	0.130	0.096	0.253	0.028	0.074	0.000	0.006	0.264	0.082	0.041	0.086
R adTP	0.085	0.128	0.112	0.220	0.017	0.045	0.000	0.009	0.089	0.014	0.000	0.072
E adTP	0.037	0.075	0.052	0.163	0.000	0.012	0.000	0.015	0.005	0.000	0.013	0.035
Z adTP	0.030	0.103	0.051	0.148	0.000	0.013	0.000	0.004	0.011	0.000	0.000	0.034
Pto adTP	0.000	0.005	0.000	0.000	0.000	0.000	0.000	0.000	0.000	0.000	0.000	0.001
Trd adTP	0.005	0.015	0.000	0.000	0.000	0.000	0.000	0.000	0.000	0.000	0.000	0.003
RFB adTP	0.007	0.000	0.000	0.000	0.000	0.000	0.000	0.000	0.000	0.000	0.013	0.003
Number of prisons with delivery of HIV drugs	20	7	1	6	3	12	2	7	3	5	3	69
non-TCM DDD_cum_	42,224	32,784	5060	11,950	3060	24,770	4230	3000	2580	5850	840	136,348
TCM DDD_cum_	18,900	14,520	2460	6210	660	11,236	1980	960	1290	2730	510	61,456
non-TCM DDD_d_	93	72	11	26	7	54	9	7	6	13	2	299
TCM DDD_d_	41	32	5	14	1	25	4	2	3	6	1	135
Ratio non-TCM/TCM	2.2	2.3	2.1	1.9	4.6	2.2	2.1	3.1	2.0	2.1	1.6	2.2
HIV adTP	0.348	0.759	0.941	0.837	0.110	0.478	0.505	0.060	0.145	0.446	0.063	0.394
Number of prisons with delivery of HCV drugs	17	6	1	2	1	9	1	8	5	3	3	56
PEG-IFN α-2a DDD_cum_	3724	1463	1547	1253	0	2198	959	987	1078	1260	448	14,917
PEG-IFN α-2b DDD_cum_	392	140	0	0	182	315	0	1925	0	7	0	2961
Total PEG-IFN α DDD_cum_	4116	1603	1547	1253	182	2513	959	2912	1078	1267	448	17,878
BOC DDD_cum_	70	84	56	0	0	0	0	245	0	0	0	455
TVR DDD_cum_	147	49	0	0	0	455	0	0	84	84	49	868
PEG IFN α-2a DDD_d_	8	3	3	3	0	5	2	2	2	3	1	33
PEG IFN α-2b DDD_d_	1	0	0	0	0	1	0	4	0	0	0	6
Total PEG-IFN α DDD_d_	9	4	3	3	0	6	2	6	2	3	1	39
BOC DDD_d_	0	0	0	0	0	0	0	1	0	0	0	1
TVR DDD_d_	0	0	0	0	0	1	0	0	0	0	0	2
PEG-IFN α adTP	0.076	0.084	0.592	0.169	0.030	0.107	0.245	0.183	0.121	0.207	0.056	0.115
BOC adTP	0.001	0.004	0.021	0.000	0.000	0.000	0.000	0.015	0.000	0.000	0.000	0.003
TVR adTP	0.003	0.003	0.000	0.000	0.000	0.019	0.000	0.000	0.009	0.014	0.006	0.006
Number of prisons with delivery of OST	7	6	1	5	3	13	0	6	5	5	5	56
Buprenorphine DDD_cum_	119	10,738	0	154	2142	910	0	434	3073	1225	1460	20,255
Buprenorphine/Naloxone DDD_cum_	756	3598	0	0	0	3248	0	175	77		119	7973
Levomethadone DDD_cum_	222	18,556	222	0	1172	48,937	0	1444	6182	16,512	3043	96,292
Methadone DDD_cum_	2033	27,967	20,411	47,472		98,590	0			19,579		216,052
Total OST DDD_cum_	3131	60,859	20,633	47,626	3314	151,684	0	2053	9332	37,316	4622	340,571
Buprenorphine DDD_d_	0	24	0	0	5	2	0	1	7	3	3	44
Buprenorphine/Naloxone DDD_d_	2	8	0	0	0	7	0	0	0	0	0	17
Levomethadone DDD_d_	0	41	0	0	3	107	0	3	14	36	7	211
Methadone DDD_d_	4	61	45	104	0	216	0	0	0	43	0	474
Total OST DDD_d_	7	133	45	104	7	333	0	5	20	82	10	747
Buprenorphine adTP	0.002	0.562	0.000	0.021	0.357	0.039	0.000	0.027	0.345	0.200	0.182	0.130
Buprenorphine/Naloxone adTP	0.014	0.188	0.000	0.000	0.000	0.138	0.000	0.011	0.009	0.000	0.015	0.051
Levomethadone adTP	0.004	0.970	0.085	0.000	0.195	2.080	0.000	0.091	0.695	2.700	0.378	0.618
Methadone adTP	0.037	1.463	7.812	6.399	0.000	4.191	0.000	0.000	0.000	3.202	0.000	1.386
OST adTP	0.058	3.183	7.897	6.419	0.552	6.448	0.000	0.129	1.048	6.102	0.575	2.184

### TB treatment

About 41% of the 97 prisons were supplied with medicines against TB. There was no TB medicine supply at all to prisons in Saarland. Both marker substances E and Z for the intensive phase were delivered to all investigated FS except for Mecklenburg-Western Pomerania, Schleswig-Holstein and Thuringia. Both marker substances H and R for the continuation phase and chemoprevention were supplied to all FS except Thuringia. Mecklenburg-Western Pomerania and Schleswig-Holstein only received the marker substances H and R, Thuringia only the substances E and H. Substances for the treatment of drug resistant or complicated or severe TB were provided in the FS Bavaria, Berlin and Thuringia.

The adTP of E and Z in the initial stage ranged from 0% in Mecklenburg-Western Pomerania, Saarland, Schleswig-Holstein and Thuringia (Z) to 0.16% (E) and 0.15% (Z) in Hamburg. The adTP of H and R in the continuity stage ranged from 0% in Saarland and Thuringia (R) to 0.26% in Saxony-Anhalt (H) and 0.22% in Hamburg (R). In total, twice as many delivered marker substances were indicated for the continuation phase and chemoprevention than the intensive phase (H & R: 0.09% & 0.07% vs. E & Z: 0.04% & 0.03%). The formulation 400 mg isoniazid per pill played only a marginal role in Lower-Saxony, Saxony-Anhalt and Schleswig-Holstein. Pto as a marker substance for MDR-TB treatment was only delivered in Berlin (adTP 0.01%). Trd also as a marker substance for MDR-TB was delivered in Bavaria (adTP 0.01%) and Berlin (adTP 0.02%). Rfb as marker substance for the TB treatment of patients with HIV-TB-coinfection was delivered in Bavaria (adTP 0.01%) and Thuringia (adTP 0.02%).

### HIV treatment

Overall, 71% of the included prisons in the respective FS were delivered with drugs for HIV treatment. HIV DDD_cum_ ranged from 510 in Thuringia to 18.900 in Bavaria. HIV DDD_d_ ranged from 1 in Thuringia to 41 in Bavaria. HIV adTP ranged from 0.06% in Saxony to 0.94% in Bremen. The overall HIV adTP was 0.39%.

Nucleoside reverse transcriptase inhibitor (NRTI) substances and protease inhibitor (PI) substances were supplied to all participating FS. With the exception of Thuringia, all other FS were supplied with non-nucleoside reverse transcriptase inhibitor (NNRTI) substances and the integrase inhibitor (INI) raltegravir. The entry inhibitor (EI) maraviroc was supplied exclusively to Bavaria and Berlin.

### HCV treatment

In total, 58% of the represented prisons were delivered with drugs for HCV treatment. In the FS of Bremen and Saxony-Anhalt, all prisons were supplied with HCV drugs. HCV DDD_cum_ ranged from 182 in Mecklenburg-Western Pomerania to 4.116 in Bavaria. HCV DDD_d_ ranged from 0 in Mecklenburg-Western Pomerania to 9 in Bavaria. HCV adTP ranged from 0.03% in Mecklenburg-Western Pomerania to 0.59% in Bremen. The overall HCV adTP was 0.12%. BOC DDD_cum_ ranged from 0 in seven FS to 245 in Saxony. BOC DDD_d_ ranged from 0 in seven FS to 1 in Saxony. BOC adTP ranged from 0% in seven FS to 0.02% in Bremen. TVR DDD_cum_ ranged from 0 in five FS to 455 in Lower-Saxony. TVR DDD_d_ ranged from 0 in five FS to 1 in Lower-Saxony. TVR adTP ranged from 0% in five FS to 0.02% in Lower-Saxony.

### OST

Regarding opioid substitutions, 58% of the included prisons in the respective FS were supplied with drugs for OST. OST DDD_cum_ ranged from 0 in Saarland to 151.684 in Lower-Saxony. OST DDD_d_ ranged from 0 in Saarland to 333 in Lower-Saxony. OST adTP ranged between 0% in Saarland and 7.90% in Bremen. The overall OST adTP was 2.18%.

## Discussion

The results show that medical treatment of all investigated diseases took place in German penal institutions. However, under the assumption that the number of adTP corresponds to the number of treated people per day, differences in quantity and extent of treatment were observed among the FS. To what extent requirements and directives of the MoJ affect the initiation of treatment and health care seems to differ not only among FS but also among prisons within the FS [35, 42, 43]. The differences regarding treatment of diseases and OST in prisons might reflect the decentralized federal system in Germany, in which the states may pursue different approaches with respect to the management of medical care [5, 6, 39].

### TB treatment

Our data suggests intensive and continued tuberculosis treatments as well as chemoprevention in prisons of all participating FS except Saarland, where no TB medicine supply was observed. The treatment of resistant, complicated or severe TB was carried out in the FS Bavaria, Berlin and Thuringia. The federal city-states Berlin, Bremen and Hamburg showed high treatment prevalences for all TB substances, which implies largely initiated and continued TB treatment in those penal institutions. In Mecklenburg-Western Pomerania, Saarland and Schleswig-Holstein TB treatment was not initiated since these FS were not supplied with E and Z. However, Mecklenburg-Western Pomerania, Lower-Saxony, Saxony-Anhalt and Schleswig-Holstein showed high adTP of H and R which suggest mostly continued TB treatment and chemoprevention. In addition, Thuringia showed solely high H adTP, which might also indicate chemoprevention. A further indication of H is the treatment of R-resistant TB. However, since the proportion of corresponding E and Z is too low in the respective FS this may play only a marginal role [26]. Further, we observed a ratio of the marker substances for the intensive and continuation phase that might indicate incomplete standard six-month regimen in most FS. However, since imprisonment can begin or end during the course of a treatment, our observation period did not necessarily capture the entire treatment time.

In all penal institutions in Berlin, each newly incarcerated person is screened for TB by chest x-ray [24]. This active case-finding when entering prison can be equated with the prevalence of TB at the time of imprisonment [24]. Bös and Hauer found a TB prevalence of 0.11% through active case-finding by chest x-ray examinations in 2007–2010 in Berlin’s penal institutions and 0.21% in 1996–1998 [24, 44]. Our work found an adTP in Berlin of 0.08% and 0.10% for the marker substances E and Z, respectively. Comparing the most recent TB prevalence seen in Berlin’s penal institutions to the adTP from our analysis, treatment rates for TB were at least in Berlin consistent with the expected TB prevalence. The most important reason for not treating a prisoner in the study by Bös et al. was a too short duration of imprisonment [24]. We found an almost equal distribution of the adTP for H and R possibly explained by the active case-finding in Berlin with no need for chemoprevention.

Treatment of Multidrug-Resistant-TB and of complicated or severe TB were each observed in only two FS, Berlin and Bavaria and Thuringia and Bavaria, respectively. This was possibly due to a transfer of patients to prison hospitals with necessary existing technical and logistical conditions.

The large range of the provision of drugs for TB treatment among the FS could be explained by co-operations between FS. Especially the co-operation of Saarland with Bavaria and North Rhine-Westphalia might be the reason why there was no TB treatments at all supplied to prisons in Saarland. According to this arrangement Saarland transferred TB infected male prisoners to Bavaria and TB infected female prisoners to North Rhine-Westphalia for treatment. Also Thuringia had a co-operation with Bavaria and transferred TB infected prisoners to Bavaria. However, TB treatments were still carried out in Thuringia with H, E and Rfb, and the latter is indicated for HIV-TB-coinfected patients. We speculate the reason why TB treatments were still carried out in Thuringia despite existing co-operations could be overcrowding or other factors that would need further investigation.

### HIV treatment

HIV treatments were carried out in prisons of all participating FS, with highest treatment prevalences found in the federal city-states Bremen, Hamburg and Berlin. The higher HIV adTP compared to the HCV adTP is remarkable, especially considering that studies found a much lower HIV prevalence compared to the HCV prevalence among prisoners. Radun et al. found an HIV antibody prevalence of 0.7% [3]. Schulte et al. came to an HIV prevalence of 1.2%. In this study, 147 prisoners were treated against HIV per year corresponding to 1.0% of all represented prisoners and to 89% of the infected prisoners [5]. In a study by Reimer et al., 300 prisoners were treated corresponding to about 1.0% of the represented prisoners and to about 94% of the infected prisoners [39]. Our results are in accordance with these previous studies, and the treatment prevalence of 0.39% for HIV matches more or less the expected prevalence of infection. HIV treatment seems to be the only of the four investigated treatments that is offered to an adequate proportion of estimated infected prisoners.

The combination of the agents and drug classes suggest treatment according to treatment guidelines which recommend a combination of two NRTI with either a NNRTI, PI or INI for first line therapy. The proportion of NRTI DDD_cum_ to total NNRTI, PI and INI DDD_cum_ suggests standard regimen distribution. Additionally, within the drug classes the DDD_cum_ of the drugs correspond to a standard regimen. Substances differing from the standard therapy were rarely administered. This applies, for example, to the older NRTI substance didanosin and the nowadays less frequent PI substance fosamprenavir. On the other hand, newer substances were also prescribed rather infrequently, which could indicate a hesitation to apply them. This is clearly seen in rilpivirin in the substance group of the NNRTI. Furthermore, we found indication for continuation and switch of ART of previously treated prisoners. This can be seen through the delivery of etravirin in Bavaria, Hamburg and Saarland, which is indicated for the treatment in antiretroviral treatment-experienced patients.

### HCV treatment

Our data suggest that HCV treatments were provided in prisons of all participating FS. Overall, during the observation period, only 0.12% of prisoners were treated per day with HCV antivirals. This HCV treatment prevalence appears to be too low considering that studies have shown HCV prevalences to be about 14% to 21% among prisoners [3, 5, 39]. In the comparison of the FS, Bremen showed the highest HCV treatment prevalence, followed by Saarland and Schleswig-Holstein. In the two other federal city-states Berlin and Hamburg very low HCV treatment prevalences were observed, which is not consistent with the high HIV treatment prevalence in both cities. The one third lower adTP in Berlin compared to the overall adTP was therefore surprising considering Berlin has the highest incidence of newly diagnosed HCV of all FS [45], and risk group populations are disproportionately present. We assume that the prevalence of HCV and the need of treatment among prisoners differ from prison to prison depending on the proportion of prisoners from FS with higher HCV prevalence, the proportion of PWID among prisoners, as well as the proportion of prisoners originating from countries with high HCV prevalence. Also, intra- and extramural co-operations among FS may at least partially explain the different treatment prevalences [46]. Although studies found a much higher HCV prevalence than HIV prevalence among prisoners [3, 5, 39] the amount of HCV treatment per prisoner is much lower than of HIV treatment.

Furthermore, the observed HCV treatment prevalence in view of the high HCV antibody prevalence of 20.6%, 14.3% and 15.0% found among prisoners in surveys is much too low [3, 5, 39]. These studies in German prisons found low HCV treatment rates and support our findings, only 111 (0.8% of the represented prisoners) and 400 (1.4% of the represented prisoners) prisoners were treated per year [5, 39]. According to Schulte et al., the main exclusion criteria for HCV treatment were short duration of imprisonment and drug abuse [5]. Also in comparison to the prevalence of injecting drug use (IDU) by Radun et al. (29.7%) and Schulte et al. (21.9%), the HCV adTP of 0.12% appeared to be too low considering that studies have shown HCV antibody prevalences of 57.6% among PWID [3, 5, 27]. Furthermore according to current guidelines, IDU is no contraindication for HCV therapy [27].

At the time of the analysis, HCV was treated in particular with a dual combination of PEG-IFN and RBV according to the respective guidelines at that time. There was also the option of a triple therapy with one of the two protease inhibitors BOC or TVR in combination with PEG-IFN and RBV. However, this treatment was cost extensive and rich in side effects and assumedly therefore played virtually no role for the HCV treatment in prisons. Triple therapies containing BOC or TVR accounted for only 7.8% of all HCV treatments. Sligthly more triple therapies were observed in Berlin and about two times more in Bremen, Lower Saxony and Saxony. In 2013, new promising direct-acting antivirals (DAAs) against HCV had already been announced. It is possible that the low treatment numbers are partially related to the awaiting of upcoming treatment options as an analysis of drug prescription data of the general German population also suggests [47]. Furthermore, due to the relative ineffectiveness and often serious side effects of interferon-based treatment, it seems plausible that prisoners are even less likely to wish to undergo debilitating treatment than non-prisoners. However, it is unknown to what extent costly DAA regimens have been prescribed since 2014 in the prison setting. An investigation of that would be a valuable follow-up assessment of the extent and quality of medical treatment in German prisons.

### OST

We found a large range of the OST adTP between 0% in Saarland and 7.9% in Bremen. Thus, in some FS OST seems to be provided to a high proportion of prisoners, indicating a more liberal and harm-reduction-led politic. In the northern FS more prisoners had access to OST compared to Saarland and Bavaria and the eastern FS [46]. None of the prisons in Saarland and only seven penal institutions in Bavaria were supplied with OST medicines. The amount of OST doses suggests therapy in the northern FS and an abstinence and denial approach in Saarland, Bavaria and the eastern FS. This imbalance and therapy slope among the FS was already described by Keppler et al. [33, 46].

The low number of OST-supplied penal institutions in Bavaria is remarkable. Although OST needs no special medical tools or rooms and is simple to carry out only 7 of the 36 prisons were supplied with OST substances in Bavaria, corresponding to an OST adTP of 0.06%. Due to this low OST adTP, we assume a practice of denial or withdrawal rather than substitution treatments offered to prisoners in Bavaria [48]. The number of 133 DDD_d_ OST we found in Berlin correlates well with the number of 154 and 120 OST reported for Berlin prisons by Keppler in Lehmann et al. [46] and by Jakob et al. [35]. According to this, 3.6% of the prisoners in Berlin received OST compared to 3.2% in our study [46]. Schulte et al. accounted for 1,137 OST per year altogether, which corresponds to 8.0% of the represented prisoners and to 37% of the PWID in prison [5]. In Reimer’s work, 320 long time opioid substitutions correspond to about 1.1% of the represented prisoners [39]. The overall OST adTP of 2.18% we found in our study approximately matches the OST treatment prevalence of Schulte et al. and Reimer. However, given the IDU prevalence of 29.7% and 21.9% among prisoners found in other studies [3, 5], even in the FS with a comparably high OST prevalence it can be concluded that only a minority of prisoners in need receive OST. Reporting on the prevalence of opioid dependence among people in prison was recently implemented in Germany, but the data is not yet published. It might be assumed that IDU mostly consists of opioid consumption. It is possible that some people coming into prison want to use the opportunity to be treated and to stop injecting but that others might prefer a cold withdrawal or do not want to reveal their addiction to avoid stigmatization or disadvantages concerning their prison conditions. Nonetheless our data show a need for scaling up OST, at least in some of the FS.

About 25% of the male and 50% of the female prisoners in Germany are PWID [33]. OST provided during incarceration reduces the level of IDU in prison and thus the possibility of HIV and HCV transmission via unsafe use [49]. OST as an approved effective therapy functions well in a prison setting, e.g. supervised application, regularity of intake and structured daily life [33]. OST, particularly in combination with other harm-reduction strategies, is an evidence-based measure of HIV and HCV prevention [16, 50, 51]. In addition, people who receive OST often show an increased compliance regarding antiviral and antiretroviral treatment [52, 53]. For the above mentioned reasons and its protective effects, it is incomprehensible that OST is not offered in every prison. According to information provided by several prison doctors a certain proportion of PWID and thus, people in potential need of OST, are among every prison population, and no distribution of PWID to special prisons takes place. Further, this would not explain the high range of OST among the FS, suggesting an abstinence-oriented and denial approach in some FS.

IDU in prison is often unsafe due to the unavailability of sterile materials and is therefore one of the main transmission routes and major risks for HCV. Studies have shown an HCV antibody prevalence of 57.6% among PWID [3] therefore, the HCV adTP of 0.12% appeared to be too low compared to the OST adTP of 2.18%. Studies revealed that OST access depended mainly on substitution treatment before imprisonment, short duration of imprisonment and co-morbidity such as infectious diseases [5, 42].

Although OST guidelines exist for Germany [54], this work shows that these guidelines are not consistently applied, and that intramural OST highly differs among the FS and prisons. This might be due to the lack of nationwide OST guidelines for prisons [35]. However, in the absence of prison-specific guidelines the existing national OST guidelines should be applied to prisoners as well.

#### Limitations

The following limitations have to be considered in the interpretation of the data.

The evaluation of pharmacy delivery data allows no statement about which and how many medicines actually reached the individual patient. This can potentially lead to an overestimation of the calculated DDD for all evaluated drugs because they can be ordered in advance. On the other hand, emergency or ad hoc-orders are taken over by local pharmacies not included in our analysis, leading to a potential underestimation of the data and the corresponding treatments. However, according to a prison-supplying pharmacy, emergency orders amount to less than 2% [55]. Furthermore, one drug package might be used for several patients. Usually, the pills are packaged according to the prescription per patient or per patient and day [56]. We tried to avoid a bias by calculating the treatment prevalence per day. Tablets are divided only in particular cases. However, this procedure can differ from prison to prison. In addition, there are differences in the treatment management and the supply of medication in case of transfers of prisoners. In some cases, medicines are completely provided by the previously responsible prison. In other cases, after the transfer to another prison, the medicines are provided by the new prison [56].

The treatment success and failure, including side effects and drug interactions, remain unknown. We had no knowledge of the treatment duration. Therefore we calculated the average treatment prevalence as point prevalence in percent at each single day of the whole study period. For OST we did not consider initial dosage or gradual reduction of OST, but assumed a steady dosage, so we might have underestimated the number of persons under OST medication.

Because of the missing pharmacy data of one sick ward in a prison in Mecklenburg-Western Pomerania with five beds and one correctional hospital in Lower Saxony with 52 beds, the data of Mecklenburg-Western Pomerania and Lower Saxony are not complete. Therefore the DDD and adTP in these FS might be underestimated.

Several co-operations exist among the FS limiting the representativeness of the data for the respective FS. For example, Saarland had a contract to transfer ill prisoners to Rhineland-Palatinate [36]. Schleswig-Holstein had a transfer co-operation with Hamburg [37]. Thuringia had co-operations with Saxony, Saxony-Anhalt and Hessen to transfer ill prisoners [38]. Therefore the DDD and adTP of Saarland, Schleswig-Holstein and Thuringia are potentially underestimated and of Hamburg, Saxony and Saxony-Anhalt are potentially overestimated.

A further limitation is the different temporal units of the pharmacy delivery data on the one hand (per quarter of a year) and the number of the prisoners on the other hand (four calendar months). The actual duration of imprisonment as well as the information on releases such as the day of the release and the number of released prisoners cannot be derived from the available data and remain unknown. Therefore we chose to account the DDD for each day in the study period.

Moreover, this paper describes merely the proportion of treated persons among all prisoners and not among infected prisoners. To evaluate our treatment prevalence, we compared it with the prevalence seen in previous studies.

## Conclusions

This work is the first attempt to describe and assess the medical care of TB, blood-borne and sexually transmitted infections and OST in prison. The study indicates that treatment of TB, HCV, HIV and opioid dependence is carried out in German penal institutions, and that guideline-recommended substances and standard treatments are used. However, a high variation of treatment per prison population was observed among the FS and among the respective diseases, which is not fully explained by the described transfer co-operations. Providing treatment of chronic infections and OST to prisoners seems to be dependent on structural and individual factors, e.g. the prison’s medical service structure, the political attitude and the allocation of financial budget to medical treatment in the respective prison and in the FS. The WHO recommendations and the UN’s Mandela Rules maintain that prisoner health care should be consistent with the community standards of care, and under the direction of the ministry of health [57]. According to our findings, prison health care and policy in Germany is not fully consistent with this, especially with regard to treatment of HCV and OST. Treatment rates for TB were consistent with the expected TB prevalence, at least in Berlin. Treatment for HIV seems to be the one that is offered to a more or less adequate proportion of estimated infected prisoners in the FS. In the view of the expected high HCV prevalence among prison populations and in comparison to HIV and opioid dependence treatment prevalence, the HCV treatment prevalence we observed was too low. HCV treatment with DAAs has improved remarkably since the study period and will hopefully have an impact on the treatment prevalence in prisons despite high costs. Despite a varying proportion of PWID among prisoners and limitations due to a purely secondary data analysis, the large differences among the FS regarding all infection treatments and OST point to inconsistent treatment practices although nationwide extramural treatment guidelines for Germany exist. It is alarming that some FS seem to provide OST at a very low level. However, in some FS our data suggest that a high proportion of prisoners is covered with OST.

Despite its challenges, the prison setting is an opportunity for prevention and treatment of TB, HIV, HCV and OST [18] which could be carried out at a greater extent and more consistently. The regulated environment offers good requirements for e.g. distribution of sterile injection utensils, supervised application, regularity of intake and the opportunity for restructuring of daily life. Prisons therefore provide both risks for the spread of diseases but also many opportunities for prevention of these infections [17]. Continuous analyses for longer periods are necessary in order to make further statements regarding the health care situation in German prisons. A monitoring and reporting system of infectious diseases among prisoners would help to ensure equal access to treatment and to harmonize strategies among FS. Finally, correctional facilities should consistently implement prevention and harm-reduction measures such as needle-exchange and condom distribution programs to avoid further spread of diseases [30].

## Abbreviations

3TC: Lamivudine
adTP: Average daily treatment prevalence
ART: Antiretroviral therapy
ATC: Anatomical Therapeutic Chemical
BOC: Boceprevir
DAA: Direct-acting antiviral
DDD: Defined daily dose(s)
DDD_cum_: Cumulative number of DDD
DDD_d_: Average daily number of DDD
E: Ethambutol
EI: Entry inhibitor
FS: Federal state(s)
FTC: Emtricitabine
H: Isoniazid
HCV: Hepatitis C virus
HIV: Human immunodeficiency virus
IDU: Intravenous drug use
INI: Integrase inhibitor
MDR-TB: Multidrug-resistant-TB
MoJ: Ministry of Justice
NNRTI: Non-nucleoside reverse transcriptase inhibitor
NRTI: Nucleoside reverse transcriptase inhibitor
OST: Opioid substitution treatment
PEG-IFN α-2a: Peginterferon α-2a
PEG-IFN α-2b: Peginterferon α-2b
PEG-IFN: Peginterferon
PI: Protease inhibitor
Pto: Protionamide
PWID: People who inject drugs
PZN: Central pharmaceutical number
R: Rifampicin
RBV: Ribavirin
Rfb: Rifabutin
SGB: Social Act
SHI: Statutory health insurance
StVollzG: Prison Act
TB: Tuberculosis
TCM: Thiacytidine medication
Trd: Terizidone
TVR: Telaprevir
Z: Pyrazinamid
